# Placebo by proxy expectations toward acupuncture change over time: a survey comparing parental expectations to acupuncture pre- and postoperatively

**DOI:** 10.1186/s12906-018-2248-z

**Published:** 2018-06-14

**Authors:** Ingrid Liodden, Are Hugo Pripp, Arne Johan Norheim

**Affiliations:** 1 Faculty of Health Sciences, Oslo Metropolitan University, 0130 Oslo, Norway; 2 National Research Center in Complementary and Alternative Medicine, Department of Community Medicine, Faculty of Health Sciences, UiT, The Arctic University of Norway, 9037 Tromsø, Norway

**Keywords:** Expectations, Placebo by proxy, Pre- and postoperatively, Acupuncture

## Abstract

**Background:**

Patients entering a treatment have expectancy to outcome based on their previous experience, the information received, and the credibility of the treatment. Once the treatment has started, patients may detect and interpret contextual cues and somatic state. Influenced and conditioned by positive or negative interpretations, their reappraisal may improve or worsen the treatment outcome. The aims were to investigate whether parental pre-treatment expectancies towards acupuncture differ compared to post-treatment expectancies, and assess predictors for possible change of parental expectancy. Further, we wanted to explore whether the change correlates with the treatment outcome, i.e. postoperative vomiting in children.

**Methods:**

Two hundred and eighty-two parents completed per- and 24 h postoperatively a survey on their expectancy to acupuncture treatment for alleviation of postoperative vomiting in children. The survey was embedded in a randomised controlled trial.

**Results:**

Parental expectancy to acupuncture treatment changed over time. The changes were predicted by several variables such as children’s gender, parents’ age and education, previous experiences, and assignment to treatment group. The strongest predictor was parental anxiety to their child undergoing surgery. Further, the change of parental expectancy was correlated with postoperative vomiting.

**Conclusions:**

Anxious parents are prone to change their expectancy in a positive direction during the treatment period, which in turn may improve treatment outcome. Acupuncture therapists in clinical practice should pay a special attention to the potential that lies here, and acknowledge parental anxiety as a possible facilitator, and not a barrier, to elicit placebo by proxy effects. Further research to expand the findings of the present study into other treatments is in order. Future research should also provide more knowledge about how parental expectancy changes over time, and how different factors predict and produce change of parental expectancy.

**Trial registration:**

ClinicalTrials.gov NCT01729052. Registered November 20, 2012.

**Electronic supplementary material:**

The online version of this article (10.1186/s12906-018-2248-z) contains supplementary material, which is available to authorized users.

## Background

Several psychological processes are involved in placebo effects, such as expectancy to treatment [1] and cognitive reinterpretation in the form of reappraisal, which involves “reinterpreting the meaning of affective stimuli in ways that alter [their] emotional impact” [2]. These two processes may contribute to creating succeeding biological and physiological reactions and may accordingly play important roles to the magnitude of treatment effects. Patients entering a treatment have expectancy to outcome based on their previous experience, the information received, and the credibility of the treatment effect [3]. Once the treatment has started, patients may detect and interpret contextual cues and somatic state [4]. Influenced and conditioned by positive or negative interpretations, their reappraisal may improve or worsen the outcome [5].

Placebo (effect) by proxy is a term used when a placebo effect is caused by other persons’ (e.g. parents, other family members, therapist) positive expectancy rather than the patient’s. Parents may respond emotionally when their child receive a treatment and interpret any sign as treatment response, no matter whether there is a physiological effect or other indications of improvement. Similarly, attention may selectively be given to positive changes while observed negative changes are ignored or explained away [6]. The phenomenon placebo by proxy is underappreciated and rarely discussed, although Grelotti and Kaptchuk [6] contend that placebo by proxy probably occurs as frequently as other placebo effects and may have great implications.

Placebo experts have developed a research agenda for further progress in the field. With the goal of optimizing patients’ responses to treatment, they recommend that studies should be conducted to determine how modifications in expectation (…) affect treatment outcomes ([7], p., 293). In connection with their recommendation, we have attempted to clarify some aspects of the psychological processes, which include expectancy during the clinical encounter. The study demonstrates that parental expectancy preceding an intervention of acupuncture for postoperative morbidities in children may change over time, and points out variables that predict change of expectancy, which in turn may contribute to modifications in expectations and thus affect treatment outcomes, i.e. placebo effect by proxy. The study may be of value for enhancing placebo by proxy effects in therapeutic treatments, and for implementing robust and unbiased research methods.

### Aims

The aims were to investigate whether parental pre-treatment expectancies towards acupuncture differ compared to post-treatment expectancies, and assess predictors for possible change of parental expectancy. Further, we wanted to explore whether the change correlates with the treatment outcome, i.e. postoperative vomiting in children.

## Methods

The present paper is a survey embedded in a randomised controlled trial that has been published elsewhere [8].

The aim of the randomised controlled trial was to investigate the effect of acupuncture versus usual care for nausea and vomiting after tonsillectomy with or without adenoidectomy in children 1–11 years of age. The children were preoperatively healthy, no comorbidities of importance, and at low risk of anaesthesiological complications; American Society of Anaesthesiologists Physical Status Classification System ≤2. The study was carried out at three ambulatory clinics alongside normal practice. The acupuncture was performed during anaesthesia, and the needle retention time ranged from 5 to 45 min.

The principal investigator briefed the parents in the collection of data of the participating children. The parents assessed retching and vomiting by frequency using a purpose designed form. They employed a behaviour tool, FLACC-N, to measure pain in children < 5 years of age, and they assessed pain and nausea in children > 5 years of age by using The Faces Pain Scale and the BARF nausea scale. They also reported their evaluation of the children’s experience of overall malaise as none, minimal, moderate, great, and severe. The principal researcher collected the recorded data by telephone after 24 h.

### Setting and participants

Two hundred and eighty-two parents, who consented to their child participating in the randomised controlled trial, also participated in the survey. In order to avoid any subconscious election of parent by the investigator, we invited mother to participate when both parents were present at the clinic. To prevent biases of perceived treatment, constituting parents’ beliefs about group allocation, the parents were not informed about the randomisation procedure; all parents believed that their child received acupuncture treatment. All parents received exactly the same information about the study intervention, both in the intervention and the control group. The parents followed the children into the operation theatre and left when their child was anaesthetised. The parents were not present during surgery or acupuncture delivery. Postoperatively the parents were allowed to stay with their children as soon as the child was transferred to recovery unit, and after acupuncture was accomplished. They followed their child throughout the 24 h of data collection. The data were collected during October 2012 to June 2013 at three ambulatory clinics in Norway.

### Data collection

We collected data from the children’s medical records. Further, pre- and postscores of parental expectancy were assessed peroperatively and 24 h postoperatively by means of a questionnaire (Additional file 1) constituting three sections of questions with response options on visual analogue scales (VAS) from 0 to 9. Question # 5 was 24 h postoperatively amended to “How anxious are you right now”, and question # 6 was omitted.

Section 1 assessed parental expectancy by the Credibility Expectancy Questionnaire (CEQ) [9]:

- Question # 1: How logical does acupuncture treatment seem to you?
- Question # 2: How confident do you feel that acupuncture treatment can alleviate nausea, vomiting, and pain in children? (In the present article only vomiting was analysed)
- Question # 3: How confident would you be in recommending acupuncture to a friend who suffered from similar complaints?
- Question # 4: How successful do you think acupuncture would be in alleviating other complaints?

A higher rating indicated higher credibility/expectancy. The wording of the questionnaire was, as recommended by Vincent and Lewith [10], slightly amended from the original, taking into consideration the participants (proxies) and the condition to be studied.

Section 2 assessed parent’s current anxiety regarding their child undergoing surgery [11]:

- Question # 5 How anxious are you regarding the impending surgery?

A higher rating indicated higher anxiety.

Section 3 assessed parents previous experience of acupuncture [12]:

- Question # 6: Have you or your child/family members/friends ever received acupuncture treatment? If yes, was previous acupuncture helpful to you/them?

A higher rating indicated higher usefulness.

### Evaluation of questionnaires

The CEQ assessment tool was developed by Borkovec and Nau [9] and validated for use in acupuncture by Vincent [13]; the psychometric evaluation showed an adequate internal consistency and test-retest reliability in a clinical sample. A Norwegian translation of all questions was back-translated by a bilingual scholar for the verification of the quality. Cognitive interviews showed that VAS for anxiety evaluation was well understood and easily completed by patients with anxiety. Test-retest reliability was adequate [11]. Another validation of VAS concluded that VAS was a reliable indicator of preoperative anxiety, when tested at only one point of time [14]. The accuracy and appropriateness of the question about previous experience with acupuncture has been verified by specialists within different medical and psychological disciplines [12].

### Outcome measurements

Main outcome measures for the present study were parental expectancy pre- and postscores. Prescores were assessed preoperatively and postscores were assessed 24 h postoperatively. Secondary outcomes were parental anxiety and previous acupuncture experience, prior to their children’s pending surgery.

## Statistical analyses

Descriptive statistics with number of subjects and percentage or mean, median and standard deviation described continuous or categorical data, respectively. Change from pre- to postscore of parental anxiety to acupuncture was statistically assessed by paired samples t-test. To assess whether baseline characteristics predicted change of parental expectancy, we first performed a univariable linear regression analysis, and then a stepwise multivariable linear regression with backward selection with *p*-value > 0.20 as criteria for removal from the model. Backward selection starts with elimination of the least significant candidate predictor from a full model including all candidate predictors. Effect of postoperative vomiting on parental expectancy change was assessed by both a univariable regression model (unadjusted analysis) and a multivariable linear regression model adjusted for possible confounders.

## Results

Baseline characteristics are shown in Table 1. Of significance is the small number of fathers participating as compared to mothers, the high frequency of parents’ education more than 12 years, and that parental anxiety mean and median were above the scale’s average 4.5 score.

**Table 1:** Descriptive frequency analysis on baseline characteristics

Characteristics	*n* (%)
Mother participating	245 (86,9)
Age parents mean/median (SD)	36/36 (5534)
Female child	163 (57.8)
Age children mean/median (SD)	4/4 (2499)
Parent’s education < 12 years	79 (28)
Health professional parents	100 (35.5)
Parental anxiety mean/median (SD)	5.23/6.00 (2.809)
Previous acupuncture useful	133 (47.2)
Assignment to acupuncture	138 (48.9)

Values are numbers (%) unless stated otherwise. *N* = 282

### Parental mean change of expectancy

Parents overall mean pre- and post-scores of the four variables of expectancy to acupuncture (question 1 to 4) were above the scale’s average 4.5 score.The paired t-test showed that three out of the four variables changed during 24 h (from pre-test to post-test). Parents’ opinion of how *logical* acupuncture seemed, had decreased in the post-test. Parents were more confident that acupuncture can *alleviate* vomiting, and that they would *recommend* acupuncture to a friend. There was no change of how successful they considered acupuncture to be in alleviating other complaints (Table 2).

**Table 2:** Paired samples statistics and paired t-test

	Mean pre-score (SD)	Mean post-score (SD)	Mean difference (SD)	CI 95%	*P*-value
Question # 1 *Logical*	7,02 (1725)	6,76 (2254)	− 0.259 (1.931)	− 0.485 – 0.032	0.025*
Question # 2 *Alleviate*	5.21 (2.048)	5.82 (2.647)	0.610 (2.574)	0.308–0.912	< 0.001*
Question # 3 *Recommending*	6.30 (2.139)	7.41 (2.177)	1.106 (2.036)	0.868–0.345	< 0.001*
Question # 4 *Successful*	5.98 (1.848)	6.15 (1.887)	0.174 (1.667)	−0.022 – 0.369	0.081

Mean pre- and post-scores of parental anxiety to acupuncture, and mean differences between post- and pre-scores. Score options from 0 to 9, a higher rating indicated a higher credibility/expectancy. *N* = 282

*Significant: *p*-value ≤0.05

### Predictors causing change of parental expectancy

The baseline characteristics (Table 1) are possible variables for predicting change of parental expectancy from pretest to posttest. In a univariable model of regression analysis of these variables, we found that children’s assignment to acupuncture group predicted a positive change (*p* = 0.043) in of how *logical* acupuncture seemed to the parents (question # 1). A positive change of how confident parents felt that acupuncture can *alleviate* vomiting (question # 2), and of how *successful* they thought acupuncture would be in alleviating other complaints (question # 4), were predicted (*p* = 0.027 and 0.003 respectively) by an increasing parental anxiety. No predictors were found for change of how confident parents would be in *recommending* acupuncture (question # 3) (Table 3).

**Table 3:** Stepwise multivariable prediction model of regression analysis. Variables^a^ for predicting change of parental expectancy. *N* = 282

Dependent variables	Independent variables: selected predictors	B (CI 95%)	*P*-value ≤0.2
Question # 1: *Logical*	If female child	0.375 (−0.081–0.830)	0.107
	If education parents > 12 years	− 0.429 (0.933–0.076)	0.096
	Parental anxiety^b^	0.058 (− 0.023–0.138)	0.159
	If assignment to acupuncture	0.740 (0.249–1.230)	0.003
Question # 2: *Alleviate*	Parental anxiety^b^	0.121 (0.014–0.228)	0.026
	If assignment to acupuncture	0.594 (−0.135 – 1.322)	0.110
Question # 3: R*ecommending*	Acupuncture previously useful	−0.339 (− 0.826–0.149)	0.173
Question # 4: *Successful*	Age parents	− 0.062 (− 0.097 – − 0.028)	< 0.001
	If health professional parents	0.327 (− 0.068–0.722)	0.105
	Parental anxiety^b^	0.128 (0.059–0.197)	< 0.001
	Acupuncture previously useful	− 0.286 (− 0.671–0.100)	0.146
	If assignment to acupuncture	0.599 (0.001–1.197)	0.050

^a^Variables are selected predictors, according to *p*- value ≤0,2. *P* ≤ 2 is used to provide better predictions and more power for the selection of predictors ([13] p. 196)

^b^Continuous variable on a scale from 0 to 9, a higher rating indicated higher level of anxiety

Using a stepwise multivariable regression analysis, we found that parents of female children, increasing parental anxiety, and parents having children assigned to acupuncture group, predicted a positive change of how *logical* acupuncture seemed to them (question # 1). Parents with education more than 12 years predicted a negative change of their opinion on how logical acupuncture seemed, in relation to those with less education. A positive change of how confident parents felt that acupuncture treatment can *alleviate* vomiting (question # 2) was predicted by increasing parental anxiety, and children’s assignment to acupuncture group.

Parent’s previous experience of acupuncture as useful predicted a negative change of confidence in recommending acupuncture (question # 3) and of how successful parents thought acupuncture to be (question # 4), as compared to those who did not previously find acupuncture useful. As to question # 4, a negative change was predicted by young parents, and a positive change was predicted by health professional parents, increasing parental anxiety, and by allocation to acupuncture group (Table 3).

### Associations between vomiting and change of parental expectancy

A linear regression analysis showed that postoperative vomiting influenced on the changes in parental expectancy, also when adjusted for as possible confounders. The variable *Alleviate* was the most associated with vomiting, followed by *Logical, Recommending,* and *Successful*. Further, the regression analysis showed that an increase of expectancy scores from pre-treatment to post-treatment was correlated with no vomiting, and, conversely, a decrease of expectancy scores was correlated with vomiting (Table 4).

**Table 4:** Linear regression analyses

Parental expectancy change	Postoperative vomiting	B (CI 95%)	*P*-value
Logical	Unadjusted	**−**1.275 (− 1.705 to − 0.846)	< 0.001
	Adjusted^a^	− 1.271 (− 1.703 to − 0.839)	< 0.001
Alleviate	Unadjusted	**−**1.674 (−2.247 to − 1.100)	< 0.001
	Adjusted^a^	− 1.721 (− 2.300 to − 1.143)	< 0.001
Recommending	Unadjusted	−0.897 (−1.365 to − 0.429)	< 0.001
	Adjusted^a^	− 0.927 (− 1.397 to − 0.456)	< 0.001
Successful	Unadjusted	−0.736 (−1.119 to − 0.353)	< 0.001
	Adjusted^a^	− 0.777 (− 1.153 to − 0.401)	< 0.001

Unadjusted and adjusted associations between postoperative vomiting and changes of parental expectancy

^a^Adjusted for children’s and parents’ age, children’s gender, parents’ education, parental anxiety

## Discussion

This study shows that overall, parental expectancy to acupuncture treatment changed over time. We found that the variable *successful* did not significantly change between pre- and postscore (Table 2). However, the regression model for estimating change score for *successful* had several selected predictors such as children’s gender, parents’ age and education, previous experiences, and assignment to treatment group (Table 3). Even though it was not a significant mean change in the variable successful, several variables may modify the chance score. The strongest predictor was *parental anxiety* to their child undergoing surgery which increased the change score. Further, and as expected, the findings showed that the change of parental expectancy was correlated with the treatment outcome postoperative vomiting in children (Table 4).

Research on *parents*’ expectancy to treatment and the knowledge of placebo effects by proxy is sparse [6, 14]. Thus, in this discussion, we relate to research mostly based on *patients*’ expectancy and anxiety, acknowledging that there may be differences.

### Association with expectancy and treatment outcome

A systematic review by Colagiuri and Smith [15] demonstrated a significant association between patients’ expectancies and acupuncture treatment outcomes, and, as to alternative therapists, they did indeed appreciate the placebo effect as part of treatment outcome. Patients’ expectancy, modulated by contextual setting and patients’ experience during the therapy, is considered crucial to a successful treatment [16]. This notion may also be applied to parents, as their expectancy also plays an important role [17, 18]. As to the correlation between expectancy and postoperative vomiting found in the present study, probably the same mechanisms are here at play.

#### Predictors causing change of expectancy

To our knowledge, no previous study has investigated the change over time of parental expectancy. The analyses using linear regression models showed that parental anxiety was a strong predictor for a positive change of expectancy towards acupuncture treatment. One may speculate that anxious parents are more inclined to pin their faith on complementary treatment for their children, and consequently appreciate an increasing credibility of acupuncture during the treatment period. Consequently, anxious parents may seem to interpret cues and reappraise the treatment in a positive direction, which in turn may modulate and optimise placebo by proxy effects.

Research on patient anxiety related to placebo effects is equivocal. A reduction of anxiety is suggested to enhance the effect of treatment [19]. Few studies have examined the effect of anxiety specifically on acupuncture treatment outcome, but Vickland et al. [20] indicated that the anxiety level seemed to be an important factor for modulating physiological effects of acupuncture. However, Bertisch et al. [21] have contradicted this. It is important to keep in mind that previous studies have investigated patients’ *pre-treatment* anxiety. With regard to the point of time of anxiety registration these outcomes may in the present context be irrelevant either way.

Health professional parents also seem to change their expectancy in a positive direction, which may reflect health professionals’ increasing awareness and interest in CAM (complementary and alternative medicine) [22, 23]. On the other hand, parents with education of more than 12 years predicted a negative change of expectancy, i.e. their expectancy was reduced 24 h postoperatively. This is in conflict with suggestions from studies indicating that parents with higher education are more motivated to enter into CAM treatment for their children as compared to less educated parents [24, 25]. Keeping this in mind, one may theorize whether more educated parents become more attentive and conscious, resulting in a more negative reappraisal following a less positive stance when their child vomit, despite their initial motivation. This stance may also be true in older parents and parents who previously experienced acupuncture to be useful, who also displayed a negative change of expectancy.

At first glance, it seems reasonable that allocation to acupuncture group predicts change of expectancy, as found in the present study. A perceived treatment is typically related to randomised controlled trials constituting patients’ [parents’] beliefs about group allocation and is a possible source to activating or deactivating expectancy to treatment outcome [26]. However, the parents in the present study were not aware of the randomisation procedure. In that respect, this finding is difficult to interpret. It seems to have no assignable cause and may be a coincidence. We have also scrutinised the association between change of expectancy and the variable *female child*, but we have found no reasonable explanation.

#### Analytical considerations

We used a pragmatic and data-driven approach to select predictors for change of parental anxiety. Stepwise selection methods in regression analysis are frequently used in medical and clinical research. These statistical methods include only the most significant predictors in a model. As criteria for removal from the model, we used a *p*-value > 0.20. A somewhat higher *p*-value than the frequently used 0.05 in significance testing has been recommended to avoid small and under-fitted models for estimating predictions and especially for smaller datasets. Using a *p*-value as large as 0.20 or even larger may provide more power for selection of predictors with relatively weak effects and improve prediction results in smaller data sets ([13] p. 193).

#### Considerations of possible bias

It is surprising that the results showed that assignment to treatment group was a significant predictor to parental expectancy change. When analysing the data, we were not able to find any correlation, or explanation, that could be attributed to the design of the study to explain the significant association between group assignment and changes in parental expectancy. Our analysis and the subsequent discussion are based on what we found, and the study design did not reveal any obvious flaws or misconduct. It is challenging to assign the deception to play a role with regard to this association. However, we cannot rule out that an unknown confounder is at play.

Participation bias is a possible confounder in the present study and might have affected the outcomes. The parents who participated might not have been representative of the whole, relevant population, and e.g. have different perceptions and views on acupuncture effectiveness compared to those who did not participate.

Parents might have searched for so called *belief perseverance* and not admit any change of expectancy if the outcome contradicted their initial perceptions, resulting in confirmation bias. One may also speculate that parental fear of being impolite towards the researcher could influence on the results. It is possible that parents consciously or unconsciously adjusted their responses on questions about expectancy and anxiety. Thus, one cannot exclude the possibility of *eager to please*, causing a response bias. Unintentional communication problems between researcher and participants may have produced inaccurate or incorrect results resulting in questionnaire bias. We used a face-to-face survey; the parents partly completed the questionnaires under guidance from the principal researcher. This was intended to enhance data quality, as misapprehensions could be clarified. However, the researcher’s conscious or unconscious influence on the parents’ behavior might have led to experimenter bias.

An unfair amount of pressure applied to the subjects may give rise to procedural bias, and this might be the case when the parents were forced to complete their responses during an allotted time. The completion of the questionnaires was done while the child underwent surgery. Asking the parents to complete the survey during surgery was a pragmatic stance; the encounter with the researcher was limited in time, and this is the period of time when parents don’t do much but wait. However, the parents may have been preoccupied with and anxious about their child’s surgery at that stage. However, several of the parents spontaneously expressed that they were pleased to have a task during this period, making the time pass more quickly, and no parent claimed pressure or stress related to the completion of the questionnaire. Nevertheless, this may have biased the results.

All children were intubated, and the anaesthetics and postoperative medication were given at the anaesthesiologist’s discretion. The variation due to a flexible study protocol allowed the intervention to be similar to a normal clinical setting, and the variation would nevertheless have been ruled out due to the randomisation process. The parents were not present during surgery, and they were not able to observe the study intervention. Further, the great advantage in our study design is that all the parents (both in placebo and intervention group) were exposed to the same information, the same stressors and observations of their child, during pre-, per- and postoperative procedure. Therefore, it is unlikely that there should be a difference in influence between patients to children in intervention and control group. Parents having children undergoing surgery demonstrate anxiety [27]. Specifically they are anxious about surgery, anaesthesia and postoperative pain [27]. This might have biased the results. However, the anxiety levels might probably have been levelled out in the two groups.

Finally, it is important to be aware of participants’ different reasons to complete a questionnaire. It can be due to interest, boredom, and a desire to help others [28].

#### Strengths and limitations

Strengths of this study are the large sample size, and no missing data due to a meticulous follow-up by the researcher.

One limitation is the selection of variables to be considered predictors for change of parental expectancy. Type of surgery is one variable that might have been of importance and could have been included. However, in the main RCT study, there were no differences in effect of acupuncture according to surgical type. This variable was therefore not regarded to correlate with change of parental expectancy.

Another limitation of this study is the assessment of anxiety of one parent only. Involving both parents might have been an optimal choice. Another limitation is the use of VAS as the only tool for the assessment of anxiety. VAS may typically be employed along with questionnaires that are more comprehensive. Since the administration of VAS takes only a few seconds and easily allows a quantitative assessment, we took a pragmatic stance. Some caution is thus required when drawing conclusions.

## Conclusions

We suggest that anxious parents are prone to change their expectancy in a positive direction during the treatment period, which may in turn improve treatment outcome. Acupuncture therapists in clinical practice should pay a special attention to the potential that lies here, and acknowledge parental anxiety as a possible facilitator, and not a barrier, to eliciting placebo by proxy effects. Further research to expand the findings of the present study into other treatments is in order. Future research should also provide more knowledge about how parental expectancy changes over time, and how different factors predict and produce change of parental expectancy.

## Additional file

Additional file 1: Questionnaire: Parental expectancy, anxiety and previous experience with acupuncture assessed by a questionnaire constituting three sections of questions with response options on visual analogue scales from 0 to 9. (DOCX 24 kb)
