# Supplementary material for: Placebo by proxy expectations toward acupuncture change over time: a survey comparing parental expectations to acupuncture pre- and postoperatively
Source: BMC Complement Altern Med. 2018 Jun 14;18:183. doi: 10.1186/s12906-018-2248-z (PMC6001019; doi:10.1186/s12906-018-2248-z)
Supplement: Supplementary file 1 — Questionnaire: Parental expectancy, anxiety and previous experience with acupuncture assessed by a questionnaire constituting three sections of questions with response options on visual analogue scales from 0 to 9. (DOCX 24 kb) [file 12906_2018_2248_MOESM1_ESM.docx]

**Additional file 1**

Questionnaire: Parental expectancy, anxiety and experience

| **Section 1.** Parental expectancy  **Question # 1** How logical does acupuncture treatment seem to you? **Question # 2** How confident do you feel that acupuncture treatment can alleviate nausea, vomiting, and pain in children?  **Question # 3** How confident would you be in recommending acupuncture to a friend who suffered from similar complaints?  **Question # 4** How successful do you think acupuncture would be in alleviating other complaints?  **Section 2.** Parental anxiety **Question # 5** How anxious are you regarding the impending surgery?  **Section 3.** Previous experience with acupuncture **Question # 6** Have you or your child/family members/friends ever received acupuncture treatment? If yes, was previous acupuncture helpful to you/them? |
| --- |

The response options were indicated on a visual analogue scale (VAS) from 0 to 9.

In section 1, 2 and 3 a higher rating indicated higher credibility/expectancy, anxiety and usefulness, respectively.
